# Identification of a serum-based miRNA signature for response of esophageal squamous cell carcinoma to neoadjuvant chemotherapy

**DOI:** 10.1186/s12967-018-1762-6

**Published:** 2019-01-03

**Authors:** Yukiko Niwa, Suguru Yamada, Fuminori Sonohara, Keisuke Kurimoto, Masamichi Hayashi, Mitsuru Tashiro, Naoki Iwata, Mitsuro Kanda, Chie Tanaka, Daisuke Kobayashi, Goro Nakayama, Masahiko Koike, Michitaka Fujiwara, Yasuhiro Kodera

**Affiliations:** Department of Gastroenterological Surgery, Nagoya University Graduate School of Medicine, 65, Tsurumai-cho, Showa-ku, Nagoya, 466-8550 Japan

**Keywords:** Response to neoadjuvant chemotherapy, Esophageal cancer, Micro RNA, Minimally-invasive biomarker

## Abstract

**Background:**

Neoadjuvant chemotherapy (NAC) has become the standard of care for resectable esophageal squamous cell carcinoma (ESCC) which is one of the most lethal cancers, to improve resectability and prognosis. On this basis, to provide individually optimized therapy for ESCC, a minimally-invasive biomarker for response to NAC is strongly desired. This study aimed to identify the miRNA signature in serum specimens taken from ESCC patients undergoing NAC through genome-wide microarray technology.

**Methods:**

Comprehensive miRNA-expression profiles of serum specimens from ESCC patients before initial treatment were analyzed using microarray. A qPCR assay was performed to test the robustness of identified serum-based miRNA signature for discriminating response to NAC with serum specimens taken from 100 ESCC cases undergoing NAC.

**Results:**

We prioritized 62 miRNAs differentially expressed between responders and non-responders (absolute log_2_ fold change > 1.0, corresponding *P *< 0.05) and from the 62 miRNAs, we selected the miR-23a-5p, miR-193b-5p, and miR-873-3p, which were highly expressed in non-responders. Following qPCR analysis indicated the expression of miR-193b-5p and miR-873-3p in serum specimens were significantly higher in non-responders among three selected miRNAs (*P* = 0.004 and 0.001, respectively). Subsequently, we developed 2-miR-model (miR-193b-5p and miR-873-3p), 3-miR-model, and 2-miR + lymphatic invasion (ly) model based on logistic regression analysis, which achieved the better area under the receiver operating characteristic curves than those of single miRNAs as 2-miR-model, 0.70 (95% CI 0.57 to 0.82); 3-miR-model, 0.70 (95% CI 0.57 to 0.83); and 2-miR + ly, 0.73 (95% CI 0.60–0.86), respectively. Furthermore, we compared the detective power of the combined model: 2-miR + ly for discriminating non-responders to NAC, to other pretreatment clinical features. Consequently, 2-miR + ly model was superior to serum SCC antigen with great significance (P = 0.01) and to ly, and clinical T stage with marginal significance (P = 0.18, 0.07, respectively).

**Conclusions:**

Collectively, we demonstrated that the potential of a multi-miRNA biomarker for identifying NAC response in ESCC is realistic, and can be used in the clinic with the further validation.

**Electronic supplementary material:**

The online version of this article (10.1186/s12967-018-1762-6) contains supplementary material, which is available to authorized users.

## Background

Esophageal cancer (EC) comprising of two main histological subtypes, esophageal squamous cell carcinoma (ESCC) and esophageal adenocarcinoma, is one of the most deadly malignancies worldwide and is the sixth leading cause of cancer-related mortality with an estimated 400,000 deaths in 2012 [1]. ESCC accounts for 80% of all ECs, and is highly prevalent in eastern Asia, as well as eastern and southern Africa [2, 3]. Preoperative chemotherapy or chemoradiotherapy have become the standard of care for resectable ESCC to improve resectability and prognosis [4, 5] through tumor shrinkage and elimination of micro metastases [6, 7]. In Japan, neoadjuvant chemotherapy with 5-fluorouracil (5FU) and cisplatin is the current standard for clinical stage II/III ESCC reflecting results of a randomized trial comparing this strategy with postoperative adjuvant chemotherapy using the identical drug combination [4]. However, so far, an ideal biomarker to predict chemosensitivity for this combination is unavailable, and is strongly warranted in the era of precision medicine.

Micro RNAs (miRNAs) have been identified as important regulators of gene expression in tissue-specific physiological pathways, in response to environmental cues and in various diseases including human malignancies [8, 9]. The mature form of miRNA is short nucleotide molecules as 21–25 base pairs and miRNA is capable of inhibiting transcription by inducing degradation of the target mRNAs [10]. Due to their high stability both in the cell and in extracellular body fluids such as blood, urine, and saliva, miRNAs are attractive candidates for minimally-invasive biomarker of various diseases including ESCC [11–13]. Although the functional roles of miRNAs in tumor biology are incredibly complicated and all the functions have not been fully discovered, we expect that circulating blood miRNA could predict clinical behavior of ESCC including drug sensitivity.

The aim of this study was to identify the miRNA profiles in serum specimens taken from ESCC patients undergoing neoadjuvant chemotherapy (NAC) through genome-wide microarray technology. In addition, we tried to evaluate role of the selected miRNA biomarkers as a predictors of response to NAC. Through this approach, we aimed to gain deeper understanding of the mechanism of drug sensitivity that could lead to personalized medicine for ESCC patients.

## Methods

### Patients enrolled in the study

For microarray analysis and qPCR validation of identified miRNA markers, we enrolled ESCC patient cohorts treated with NAC followed by surgery, totaling 100 cases at the Nagoya University Hospital, Nagoya, Japan, between August 2007 and January 2016. Patients were also treated with postoperative adjuvant chemotherapy according to the surgeons’ discretion. NAC regimens applied to the patients in the study were the combination of either cisplatin or nedaplatin plus 5-FU or tegafur/gimeracil/oteracil (S-1). Of total 100 cases, 54 patients underwent cisplatin and 5-FU (54%), 43 patients underwent cisplatin and S-1 (43%), and 3 patients underwent nedaplatin and 5-FU treatment (3%). Primary tumor locations assessed by an esophagogram prior to initial treatment were as follows: eight in cervix (8%), six in upper thorax (6%), 57 in middle thorax (57%), 28 in lower thorax (28%), and one in abdomen (1%). All procedures associated with the study were approved by the Institutional Review Boards of Nagoya University Hospital, and all patients provided written informed consent.

Tumor tissue specimens were histologically confirmed and were classified by tumor-node-metastasis (TNM) stages before and after surgery, according to the American Joint Committee on Cancer staging handbook, 7th edition [14]. Response to NAC was determined based on the histological findings of the primary lesions after esophagectomy according to Japanese Classification of Esophageal Cancer 10th edition (Additional file 1: Table S1) [15]. The cases with Grade 2 and 3 were defined as responders and the cases with Grade 0 and 1 were defined as non-responders. All the cases underwent surgical resection after NAC and curability at the time of surgery was R0 in 95 cases (95%) and R1 in 5 (5%).

### Comprehensive analysis of miRNA expression and signature identification

To identify a miRNA signature for discriminating NAC response, comprehensive miRNA-expression profiles of pretreatment serum specimens from NAC responder (N = 4) and non-responder (N = 4) included interrogation of 2565 probes, using 3D-Gene^®^ Human miRNA Oligo Chip ver.21 (TORAY, Kanagawa, JAPAN). Total RNA was extracted from a 300 µl of serum specimen using 3D-Gene^®^ RNA extraction reagent from a liquid sample kit (TORAY, Kanagawa, Japan). Comprehensive miRNA expression analysis was performed using a 3D-Gene^®^ miRNA Labeling kit (TORAY, Kanagawa, Japan) and a 3D-Gene^®^ Human miRNA Oligo Chip (TORAY, Kanagawa, Japan). The annotation and oligonucleotide sequences of the probes were conformed to the miRBase: miRNA database release 21 (http://www.mirbase.org/). The chips were stringently washed, and fluorescent signals were scanned with the 3D-Gene^®^ Scanner (TORAY, Kanagawa, Japan) and analyzed using 3D-Gene^®^ Extraction software (TORAY, Kanagawa, Japan). The digitalized fluorescent signals provided by the software were regarded as the raw data. Individual miRNAs were regarded as present if the corresponding microarray signals were more than the mean plus 2 standard deviation of the blank spot signals of which the top and bottom 5% ranked by signal intensity were removed. Next all the normalized data were globally normalized per microarray, such that the median of the signal intensity was adjusted to “25”. All microarray data of the study are in agreement with the Minimum Information About a Microarray Experiment (MIAME) guidelines [16].

### RNA extraction and qPCR analysis

Total RNA was extracted from serum specimens using the miRNeasy Serum/Plasma Kit (Qiagen, Hilden, Germany). RNA was extracted from 200 µl serum according to manufacturer’s instructions. For miRNA-based quantitative PCR (qPCR) analysis, 10 ng of total RNA was reverse transcribed using the TaqMan^®^ Micro RNA Reverse Transcription Kit (Applied Biosystems, Foster City, CA) in a total reaction volume of 15 µl. qPCR was performed with MicroRNA Assay Kits (Applied Biosystems, Foster City, CA) and TaqMan^®^ Universal MasterMix II, no UNG (Applied Biosystems, Foster City, CA) according to the manufacturer’s instructions. Briefly, the reaction master mix containing 10 × RT buffer, 5 × RT primers, MultiScribe reverse transcriptase, RNase inhibitor, 100 mM dNTPs and nuclease-free water was mixed with 20 ng of total RNA extracted from the serum specimens. The mixtures were incubated for 30 min at 16 °C, 30 min at 42 °C and 5 min at 85 °C. The PCR was done using 10 µl of PCR master mix containing TaqMan 2 × Universal PCR Master Mix, 20 × TaqMan^®^ MicroRNA Assay Mix (Applied Biosystems, Foster City, CA) and the RT products in a volume of 20 µl. The reaction mixtures were incubated in a 96-well plate at 95 °C for 10 min followed by 40 cycles of 95 °C for 15 s and at 60 °C for 1 min using the StepOnePlus Real-Time PCR system (Applied Biosystems, Foster City, CA). The mean Ct values of each sample were determined from duplicate reactions. The relative expression level of each miRNA examined was expressed as ΔCt, which was defined as the subtraction of the Ct value of the target miRNA from the Ct value of the internal control miRNA-16 [17, 18]. The TaqMan^®^ Assays used in the study are shown in Additional file 1: Table S2.

### Statistical analyses

Continuous variables were expressed as median (range, minimum to maximum) compared using Wilcoxon signed-rank test. Categorical variables were compared using the χ^2^ or Fisher’s exact tests, as appropriate. miRNAs whose expression have absolute log_2_ fold change > 1.0, corresponding *P *< 0.05 with Student t test between responders and non-responders were selected for drawing the heatmap further assessing with unsupervised hierarchical clustering. In the validation with qPCR assay, expression of each miRNA was analyzed by a t-test and logistic regression analysis was performed to build a multivariate scoring model. The method developed by DeLong, et al. was employed to test the statistical significance of the difference between receiver operating characteristics (ROC) curves [19]. Recurrence-free survival (RFS) rates were estimated using the Kaplan–Meier method and compared using a log-rank test. We adhere to the STARD guidelines to report this study [20]. All statistical analyses were performed using R version 3.4.3 (https://www.r-project.org/).

## Results

### Identification of miRNA signature for NAC response of ESCC using a microarray-based miRNA expression profiling

**Fig. 1:**
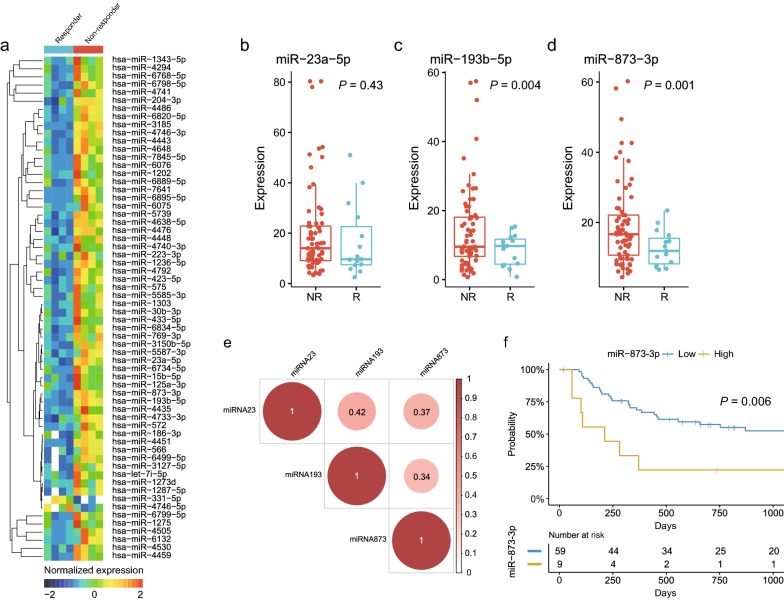
Identification of miRNA signature for response to neoadjuvant chemotherapy (NAC) through a microarray-based miRNA expression profiling. **a** A heatmap of the identified 62-miRNA signature differentially expressed between responder and non-responder to NAC. **b** miR-23a-5p expression levels in serum specimens taken from responders and non-responders to NAC. **c** miR-193b-5p expression levels in serum specimens taken from responders and non-responders to NAC. **d** miR-873-3p expression levels in serum specimens taken from responders and non-responders to NAC. **d** Correlation analysis of expression levels of miR-23a-5p, miR-193b-5p, and miR873-3p. Correlations among three miRNAs were expressed as Spearman’s rank correlation values. **e** Recurrence-free survival stratified by expression level of miR-873-3p. Statistical comparison was based on log-rank test. *R* responder, *NR* non-responder

To develop a miRNA signature for prediction of NAC response of ESCC, we first interrogated a microarray-based miRNA expression profiling results in pre-treatment serum specimens of eight ESCC patients treated with NAC consisting of cisplatin plus 5FU or S-1. Of the total 2565 miRNAs, 62 miRNAs were differentially expressed between responders and non-responders to NAC (absolute log_2_ fold change > 1.0, corresponding *P *< 0.05, Student t-test, Additional file 1: Table S3). A heatmap of the 62-miRNA signature is shown in Fig. 1a and correlation analysis among 62 miRNAs is shown in Additional file 1: Figure S1. To reduce the number of miRNAs from these differentially expressed 62 miRNAs for a clinically viable and practical assay, we selected the miR-23a-5p, miR-193b-5p, and miR-873-3p, which were highly expressed in serum from non-responders and were potentially considered having relevance to chemo-resistance by published papers, for the further validation using qPCR assay [21–23].

### qPCR verification with serum specimens taken from ESCC patients treated with NAC

Using serum specimens taken from 100 ESCC patients prior to initial treatment, we performed qPCR assay of three selected miRNAs (miR-23a-5p, miR-193b-5p, and miR-873-3p). Out of 100 ESCC cases treated with NAC from our clinical cohort, 84 cases whose qPCR results of the selected miRNAs were available were enrolled to the final analysis. The patient characteristics of these 84 ESCC cases are summarized in Table 1. There were 15 responders (18%) and 69 non-responders (72%) in this cohort. Next, when the clinical features of the 84 ESCC cases were stratified by the pathohistological response of the primary lesion to NAC, the distribution of pathological T stage was significantly different between responders and non-responders (*P* < 0.0001) whereas the distribution of pathological stage and pretreatment serum CEA value were marginally significant (*P* = 0.07, Table 2).

**Table 1:** ESCC patient characteristics (n = 84)

Characteristics		Value
Response to preoperative chemotherapy	0, 1/2, 3	69/15
Age	Years	65 (30–77)
Sex	F/M	14/70
cT	0/1/2/3/4	0/11/12/59/2
cN	0/1/2/3	10/47/23/4
cM	0/1	79/5
cStage	0/1/2/3/4	0/3/22/54/5
pT	0/1/2/3/4	4/23/19/29/9
pN	0/1/2/3	29/28/16/11
pM	0/1	79/5
pStage	0/1/2/3/4	2/17/27/33/5
Differentiation^a^	Poor/moderate/well	10/59/8
Lymphatic invasion	0/1	31/53
Venous invasion	0/1	59/25
Pretreatment serum CEA^b^	ng/ml	2.53 (1–6)
Pretreatment serum SCC antigen^c^	ng/ml	1.46 (0.5–6.7)

*ESCC* esophageal squamous cell carcinoma, *CEA* carcinoembryonic antigen, *SCC* squamous cell carcinoma

^a^There were seven cases without information of pathological differentiation

^b^There was one case without serum CEA value

^c^There was one case without serum SCC antigen value

**Table 2:** Clinical features stratified by response to preoperative chemotherapy (n = 84)

Variables	Response		*P*
	Grade 0, 1	Grade 2, 3	
Sex			
Female	12	2	1.00
Male	57	13	
Age			
Years	65 (41–77)	66 (30–76)	0.56
cT			
1	8	3	0.78
2	10	2	
3	49	10	
4	2	0	
cN			
0	8	2	0.80
1	38	9	
2	20	3	
3	3	1	
cStage			
1	2	1	0.75
2	18	4	
3	45	9	
4	4	1	
pT			
0	0	4	> 0.0001
1	17	6	
2	15	4	
3	29	0	
4	8	1	
pN			
0	24	5	0.90
1	22	6	
2	13	3	
3	10	1	
pStage			
0	0	2	0.07
1	14	3	
2	21	6	
3	29	4	
4	5	0	
Differentiation			
Well or moderate	56	11	0.340
Poor	10	0	
Lymphatic invasion			
< 2	23	8	0.250
≥ 2	46	7	
Venous invasion			
< 20	50	9	0.36
≥ 20	19	6	
Pretreatment CEA			
ng/ml	2.6 (1.0–6.0)	1.9 (0.4–4.3)	0.060
Pretreatment SCC			
ng/ml	1.1 (0.5–6.4)	1.4 (0.6–6.7)	0.350

*cT* clinical T, *cN* clinical N, *cStage* clinical Stage, *pT* pathological T, *pN* pathological N, *pStage* pathological Stage, *CEA* carcinoembryonic antigen, *SCC* squamous cell carcinoma

According to the qPCR analysis, of the 3 selected miRNAs, the expression of miR-193b-5p and miR-873-3p in serum specimens were significantly higher in non-responders (*P* = 0.004 and 0.001, respectively, Fig. 1b–d). The correlation analysis of three selected miRNAs identified the moderate linear association among them (Fig. 1e). Collectively, serum values of the three selected miRNAs were consistently higher among the non-responders, both through microarray and qPCR analyses.

### Prognostic value of the selected miRNAs’ expression

The median follow-up duration for all cases after surgery was 24 months (ranging from 21 days to 100 months). Prognostic analysis of RFS indicated that high expression of miR-873-3p in the serum specimen was associated with significantly inferior RFS (*P* = 0.006, Fig. 1f), while miRNA-23a-5p and miR-193-5p did not have significant association with RFS (miRNA-23a-5p, *P* = 0.9; miR-193b-5p, *P* = 0.91).

### Evaluation of selected miRNAs and combined models as biomarkers for NAC response

**Fig. 2:**
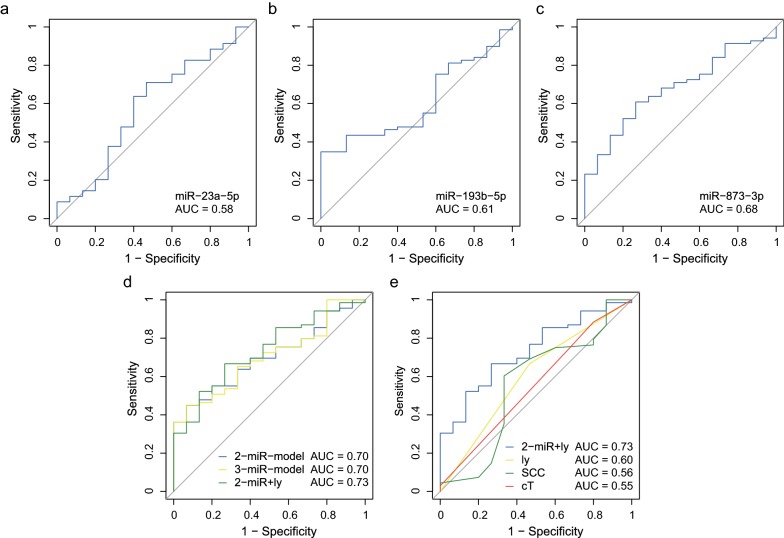
Evaluation of selected miRNAs and combined models for discriminating non-responders to NAC for ESCC. **a** A receiver operating characteristic (ROC) curve of miR-23a-5p. **b** A ROC curve of miR-193b-5p. **c** A ROC curve of miR-873-3p. **d** Comparison of ROC curves of 2-miR-model (miR-193b-5p and miR-873-3p), 3-miR-model (miR-23a-5p, miR193b-5p, and miR-873-3p), and 2-miR + ly (miR-193b-5p, miR-873-3p, and lymphatic invasion). **e** Comparison of ROC curves of 2-miR + ly, lymphatic invasion, serum SCC antigen and clinical T stage. *AUC* area under the ROC curve

To evaluate the capability of selected miRNAs as biomarkers for predicting NAC response, a binary logistic regression model was firstly applied to reveal odds ratios (ORs) for detecting non-responders to NAC, of several pretreatment clinical features such as age (≥ 65 vs < 65), sex (male vs female), clinical T stage (3, 4 vs 0, 1, 2), clinical N stage (positive vs negative), serum SCC antigen (≥ 1.5 vs < 1.5 ng/ml), and selected three miRNAs. According to the analysis with binary logistic regression models, miR-23a-5p, miR-193b-5p, and miR-873-3p had the relatively high ORs compared to other clinical features (miR-23a-5p, 2.80 [95% confidence interval: 95% CI 0.90–8.8]; miR-193b-5p, 7.47 [95% CI 0.93–60.2]; miR-873-3p, 3.11 [95% CI 0.96–10.1], Table 3). Next, we assessed the robustness of these three miRNAs for identifying non-responders to NAC using ROC curve analysis. The area under the ROC curves (AUC) of miR-23a-5p, miR-193b-5p, and miR-873-3p were 0.58 (95% CI 0.41–0.75), 0.61 (95% CI 0.47–0.74), and 0.68 (95% CI 0.54–0.81), respectively (Fig. 2a–c).

**Table 3:** Univariate binary logistic regression for preoperative chemotherapy response

Variables	OR	95% CI Low	95% CI High	*P*
Age (years)				
≥ 65 vs < 65	0.55	0.17	1.8	0.31
Sex				
Male vs female	0.73	0.15	3.7	0.70
cT				
3, 4 vs 0, 1, 2	1.42	0.43	4.7	0.57
cN				
Positive vs negative	1.17	0.22	6.2	0.85
Serum SCC antigen (ng/ml)				
≥ 1.5 vs < 1.5	0.39	0.13	1.2	0.11
miR-23a-5p				
High vs low	2.80	0.90	8.8	0.08
miR-193b-5p				
High vs low	7.47	0.93	60.2	0.06
miR-873-3p				
High vs low	3.11	0.96	10.1	0.06

*OR* odds ratio, *CI* confidence interval, *cT* clinical T, *cN* clinical N, *SCC* squamous cell carcinoma

To achieve better predictive power, we next built combined models composed of miR-193b-5p and 873-3p (termed as 2-miR-model), miR-23a-5p, miR-193-5p, and miR-873-3p (termed as 3-miR-model), and 2-miR-model with presence of microscopic lymphatic invasion of the primary lesion (ly, indicated as 2-miR + ly), using the multivariate logistic regression model as the follows: 2-miR-model, logit (probability) = − 0.23 + 0.05 × miR-193b-5p + 0.08 × miR-873-3p; 3-miR-model, logit (probability) = − 0.20 + (− 0.006) × miR-23a-5p + 0.05 × miR-193b-5p + 0.08 × miR-873-3p; 2-miR + ly model, logit (probability) = − 0.72 + 0.04 × miR-193b-5p + 0.09 × miR-873-3p + 0.84 × ly. As ly can be pathologically diagnosed using biopsy specimens and has a prognostic impact to EC patients treated with neoadjuvant therapy followed by surgery, we included this factor into the combined model [24]. Consequently, 2-miR-model, 3-miR-model and 2-miR + ly achieved the better AUCs at 0.70 (95% CI 0.57–0.82), 0.70 (95% CI 0.57–0.83), and 0.73 (95% CI 0.60–0.86), respectively. These results were superior to those achieved by quantifying any of the three miRNAs. On the other hand, there was no significant difference in the diagnostic power between any two of these three models (2-miR-model vs 3-miR-model, *P* = 0.93; 3-miR-model vs 2-miR + ly, *P* = 0.52; 2-miR + ly vs 2-miR-model, *P* = 0.46, Fig. 2d). Furthermore, we compared the predictive power of the combined model: 2-miR + ly for identifying non-responders to NAC, to other pretreatment clinical features such as ly, serum SCC antigen, and clinical T stage using ROC curve comparison (Fig. 2e). Two-miR + ly model was found to be significantly superior to the serum SCC antigen (*P* = 0.01), while being marginally superior to ly, and clinical T stage (*P* = 0.18 and 0.07, respectively).

## Discussion

In this article, we performed a comprehensive miRNA microarray-based expression profiling analysis of ESCC patients treated with NAC to establish a serum miRNA biomarker for predicting NAC response prior to the treatment. We subsequently evaluated the robustness of the combined model composed of selected miRNAs and ly to achieve better detective power for identification of the non-responders. We demonstrated that combined models (2-miR-model and 3-miR model) had better discriminative power than that of each single miRNA, and the model consisting of miR-193b-5p, miR-873-3p, and ly was superior to the pre-treatment ESCC features including single ly, serum SCC antigen and clinical T stage. Consequently, serum miRNA signature has the capability for identifying appropriate ESCC patients to be treated with NAC through less invasive liquid biopsy.

The availability of powerful and broad approaches for global miRNA characterization in circulating body fluid and simple procedure for quantifying the molecules suggests that the process for developing miRNA biomarkers will be more efficient than the process for developing traditional proteomic biomarker, which typically encounter problems at the point of antibody generation and quantitative assay development [11, 25]. In addition, the inherent regulatory function of miRNAs makes it likely that many miRNAs expressed in cancer tissue influence the biological behavior and clinical phenotype of the cancer including responsiveness to systemic chemotherapy [26]. Furthermore, very short length of miRNA is an advantage to be used as minimally-invasive biomarkers because miRNAs are expected to be less degraded in body fluid than other relatively long RNAs [27].

ESCC is one of the lethal diseases and due to the fact that lymphatic flow through the esophagus submucosal layer is abundant and multi-directional, ESCC lymphatic metastasis often spreads to lymph nodes relatively far from the primary tumor site. What is worse, esophagus is anatomically juxtaposed to the cardio-pulmonary structures and that poses difficulty in removing the locally advanced ESCC with sufficient resection margin [28]. Consequently, although esophagectomy with adequate lymph node dissection is currently the most reliable treatment for patients with Stage II/III ESCC, ESCC easily recurs to locoregional site and distant organs after surgery. To improve the efficacy of surgery and regulate the heterochronic relapse, NAC in Eastern Asia along with neoadjuvant chemoradiotherapy in the West is thought to be a standard strategy for treating resectable ESCC [29, 30]. However, clinically useful biomarkers for predicting response to chemotherapy havee not been available for ESCC. In this study, only 19% of ESCC treated with NAC was regarded as pathologically confirmed responders to NAC. In other words, almost 80% of patients unfortunately may not benefit from NAC, while all patients suffer from adverse events. Thus, in order to avoid delivering an unsuitable pretreatment for ESCC individuals, availability of a robust predictive biomarker for NAC response had been awaited. In this study, we initially elucidated three miRNAs from microarray-based miRNA expression profiles including 2565 probes. We thereafter evaluated selected miRNAs and developed combined models using the miRNAs and lymphatic invasion. While the accuracy of individual miRNA was relatively modest, the combination of two or three miRNAs revealed improved diagnostic accuracy. Collectively, we demonstrated that the potential of a multi-miRNA biomarker for identifying NAC non-responder in ESCC is realistic, and can be used in the clinic pending further validation.

The recent advancements in broad genomic and transcriptomic analysis using microarray or high-throughput sequencing procedures have resulted in molecular characterization of several cancer types [31–33]. To the best of our knowledge, this is the first study to develop a serum-based multi-miRNA biomarkers for prediction of ESCC response to NAC. Although, our results indicate that our miRNA signature can distinguish ESCC response to NAC using serum samples, there were some inherent limitations to this study. Firstly, this is a single institution study. To confirm the practical capability of discovered biomarkers, evaluation of prospectively corrected specimens in multi-institutional setting should be conducted for validation. Secondly, the post-treatment and post-surgical blood specimens for qPCR assay were unavailable in this study. The dynamics of expression level of the selected miRNAs should be analyzed in future studies to evaluate potential of the miRNAs to monitor the efficacy of ESCC treatment. Finally, considering that it is common practice to endoscopically collect biopsy samples prior to the surgery, it would have been ideal to validate our serum derived miRNA-signature also in endoscopically resected specimens to explore potential application of the elucidated miRNAs.

## Conclusions

We demonstrated that multi-miRNA models had better detective power than that of each single miRNA and the model consist of identified miRNAs and presence of lymphatic invasion was superior to the other pre-treatment ESCC features. Consequently, serum miRNA signature has the capability for identifying appropriate ESCC patients to be treated with NAC by less invasive-way with the further validation.

## Additional file

**Additional file 1: Table S1.** Pathological criteria for the effects of chemotherapy. **Table S2.** The TaqMan^®^ Assays used in this study. **Table S3.** Differentially expressed miRNA between responder and non-responder to neoadjuvant chemotherapy. **Figure S1.** Correlation analysis of expression levels of 62 miRNAs differentially expressed between responders to non-responders to neoadjuvant chemotherapy.

## Abbreviations

EC: esophageal cancer
ESCC: esophageal squamous cell carcinoma
5-FU: 5-fluorouracil
NAC: neoadjuvant chemotherapy
miRNA: micro RNA
S-1: tegafur/gimeracil/oteracil
TNM: tumor-node-metastasis
qPCR: quantitative polymerase chain reaction
ROC: receiver operating characteristics
RFS: recurrence-free survival
