# Supplementary material for: Identification of a serum-based miRNA signature for response of esophageal squamous cell carcinoma to neoadjuvant chemotherapy
Source: J Transl Med. 2019 Jan 3;17:1. doi: 10.1186/s12967-018-1762-6 (PMC6317218; doi:10.1186/s12967-018-1762-6)

Supplementary materials for

Identification of a Serum-based miRNA Signature for Response of Esophageal Squamous Cell Carcinoma to Neoadjuvant Chemotherapy

Authors:

Yukiko Niwa*; Suguru Yamada[†]; Fuminori Sonohara*; Keisuke Kurimoto; Masamichi Hayashi; Mitsuru Tashiro; Naoki Iwata; Mitsuro Kanda; Chie Tanaka; Daisuke Kobayashi; Goro Nakayama; Masahiko Koike; Michitaka Fujiwara; and Yasuhiro Kodera

Contents:

Supplementary table S1.

Pathological criteria for the effects of chemotherapy.

Supplementary table S2.

The TaqMan® Assays used in this study.

Supplementary table S3.

Differentially expressed miRNA between responder and non-responder to neoadjuvant chemotherapy.

Supplementary figure S1.

Correlation analysis of expression levels of 62 miRNAs differentially expressed between responders to non-responders to neoadjuvant chemotherapy.

Supplementary table S1. Pathological criteria for the effects of chemotherapy.

Grade 0	Ineffective		No recognizable cytological or histological therapeutic effect.
Grade 1	Slightly effective		Apparently viable cancer cells (including cells having eosinophilic cytoplasm with vacuolation and swollen nuclei) account for 1/3 or more of tumor tissue, but there is some evidence of degeneration of cancer tissue or cells.
		a.	Viable cancer cells accounting for 2/3 or more tumor tissue.
		b.	Viable cancer cells accounting for 1/3 or more, but less than 2/3, of tumor tissue.
Grade 2	Moderately effective		Viable cancer cells account for less than 1/3 of tumor tissue, while other cancer cells are severely degenerated or necrotic.
Grade 3	Markedly effective		No viable cancer cells are evident.

Supplementary table S2. The TaqMan® Assays used in this study.

Assay name	Assay ID	miRBase (v21) accession number	Chromosome location	Mature miRNA sequence
hsa-miR-23a*	002439	MI0000079	Chr.19: 13836587 - 13836659 [-] on Build GRCh38	GGGGUUCCUGGGGAUGGGAUUU
hsa-miR-193b*	002366	MI0003137	Chr.16: 14303967 - 14304049 [+] on Build GRCh38	CGGGGUUUUGAGGGCGAGAUGA
hsa-miR-873-3p	470641_mat	MI0005564	Chr.9: 28888879 - 28888955 [-] on Build GRCh38	GGAGACUGAUGAGUUCCCGGGA
hsa-miR-16	000391	MI0000070	Chr.13: 50048973 - 50049061 [-] on Build GRCh38	UAGCAGCACGUAAAUAUUGGCG

Supplementary table S3. Differentially expressed miRNA between responder and non-responder to neoadjuvant chemotherapy.

		Value after global normalization								Average			
Name	ID	Responder				Non-responder				R	NR	$Log_2(N/R)$	*P*-value
hsa-miR-3185	MIMAT0015065	241.27	234.53	223.48	194.11	474.85	531.71	428.14	546.05	223.35	495.19	1.14869	0.00004
hsa-miR-4638-5p	MIMAT0019695	83.25	72.53	105.84	70.57	380.63	365.43	248.21	301.53	83.05	323.95	1.96373	0.00006
hsa-miR-1236-5p	MIMAT0022945	47.92	33.51	37.58	44.93	195.50	233.19	171.39	126.63	40.99	181.68	2.14817	0.00007
hsa-miR-193b-5p	MIMAT0004767	40.76	40.36	29.95	28.82	91.08	128.92	106.04	93.11	34.97	104.79	1.58309	0.00011
hsa-miR-4451	MIMAT0018973	8.43	7.96	8.84	6.39	18.10	18.79	14.89	15.94	7.91	16.93	1.09859	0.00014
hsa-miR-6895-5p	MIMAT0027690	79.56	70.56	60.46	69.22	483.07	1274.79	862.17	359.90	69.95	744.98	3.41285	0.00024
hsa-miR-7641	MIMAT0029782	80.03	44.51	114.02	110.17	998.11	1561.06	967.28	501.71	87.18	1007.04	3.52996	0.00027
hsa-miR-6820-5p	MIMAT0027540	286.87	206.12	225.36	246.06	489.03	750.23	506.99	531.92	241.10	569.54	1.24017	0.00041
hsa-miR-873-3p	MIMAT0022717	41.06	21.49	16.67	21.12	122.01	155.17	104.80	85.61	25.09	116.90	2.22035	0.00048
hsa-miR-4486	MIMAT0019020	303.80	202.35	294.93	251.14	484.92	662.59	541.60	515.16	263.05	551.06	1.06686	0.00065
hsa-miR-3150b-5p	MIMAT0019226	26.14	18.17	14.27	16.03	49.49	74.14	44.14	65.44	18.65	58.30	1.64417	0.00067
hsa-miR-4746-3p	MIMAT0019881	265.60	174.40	165.05	191.50	410.45	565.53	372.04	479.04	199.14	456.77	1.19771	0.00101
hsa-miR-4435	MIMAT0018951	21.50	26.21	18.66	21.54	73.07	146.96	58.04	62.87	21.98	85.24	1.95558	0.00118
hsa-miR-6499-5p	MIMAT0025450	11.06		10.57	12.80	20.56	29.77	27.39	21.49	11.48	24.80	1.11168	0.00130
hsa-miR-423-5p	MIMAT0004748	215.41	146.10	231.37	198.32	545.06	452.18	331.85	456.94	197.80	446.51	1.17466	0.00134
hsa-miR-4443	MIMAT0018961	340.43	179.68	282.88	206.36	664.80	926.35	588.49	583.25	252.34	690.72	1.45275	0.00135
hsa-miR-6798-5p	MIMAT0027496	3037.71	2310.11	1951.95	1934.65	4274.11	6667.55	4934.52	4406.35	2308.61	5070.63	1.13514	0.00169
hsa-miR-23a-5p	MIMAT0004496	32.30	32.19	17.46	20.04	66.83	77.44	59.76	51.32	25.50	63.84	1.32406	0.00203
hsa-miR-5739	MIMAT0023116	164.24	107.81	166.45	154.89	402.83	388.64	257.48	276.73	148.35	331.42	1.15969	0.00204
hsa-miR-4448	MIMAT0018967	107.52	136.81	64.41	88.62	418.73	202.11	217.65	356.82	99.34	298.83	1.58887	0.00395
hsa-miR-7845-5p	MIMAT0030420	337.57	227.91	259.49	245.72	927.72	594.73	411.61	614.45	267.67	637.13	1.25111	0.00419
hsa-miR-572	MIMAT0003237	37.07	34.18	48.06	39.49	66.87	203.82	104.32	127.33	39.70	125.58	1.66137	0.00429
hsa-miR-4648	MIMAT0019710	392.00	150.57	208.89	158.09	623.23	848.26	544.90	478.08	227.39	623.62	1.45551	0.00554
hsa-miR-6768-5p	MIMAT0027436	5260.05	2163.06	2342.67	3798.79	17783.43	10159.78	6972.87	8523.06	3391.14	10859.79	1.67915	0.00701
hsa-miR-4459	MIMAT0018981	1162.11	483.58	603.87	675.18	3023.36	2314.93	1342.59	1594.85	731.19	2068.93	1.50058	0.00708
hsa-miR-1303	MIMAT0005891	34.22	22.27	31.12	36.59	117.67	57.24	52.66	70.23	31.05	74.45	1.26179	0.00737

hsa-miR-6834-5p	MIMAT0027568	59.18	29.36	45.23	42.41	130.84	81.88	70.28	92.48	44.04	93.87	1.09171	0.00816
hsa-miR-6076	MIMAT0023701	325.43	170.62	158.29	179.12	1028.07	608.82	366.96	434.42	208.37	609.57	1.54866	0.01021
hsa-miR-4476	MIMAT0019003	195.38	77.25	166.90	118.10	271.91	343.25	349.09	243.08	139.41	301.83	1.11444	0.01058
hsa-miR-4741	MIMAT0019871	2781.13	1356.96	1694.69	2238.50	6566.13	3607.55	3194.27	4763.33	2017.82	4532.82	1.16761	0.01146
hsa-miR-4792	MIMAT0019964	342.66	129.66	173.46	184.91	623.36	479.75	360.88	391.70	207.67	463.92	1.15958	0.01188
hsa-miR-769-3p	MIMAT0003887	33.39	20.38	30.49	42.36	100.92	62.68	44.79	100.25	31.66	77.16	1.28540	0.01306
hsa-miR-6132	MIMAT0024616	475.35	358.38	268.83	426.25	483.16	1911.31	1204.36	1151.38	382.20	1187.55	1.63558	0.01552
hsa-miR-4530	MIMAT0019069	1339.49	728.85	1321.09	845.01	3053.78	2412.51	1482.36	1872.33	1058.61	2205.25	1.05877	0.01589
hsa-miR-5587-3p	MIMAT0022290	30.99	15.23	27.53	21.17	32.27	70.70	45.52	51.23	23.73	49.93	1.07326	0.01689
hsa-miR-204-3p	MIMAT0022693	2378.08	1015.03	5619.80	2928.33	7454.94	9061.63	9099.29	7129.50	2985.31	8186.34	1.45534	0.01689
hsa-miR-4294	MIMAT0016849	6248.65	1749.86	2355.98	2693.56	14962.77	7984.00	6138.79	7293.16	3262.01	9094.68	1.47926	0.01761
hsa-miR-30b-3p	MIMAT0004589	43.91	13.82	11.98	28.74	109.69	65.20	49.22	54.95	24.61	69.77	1.50298	0.01879
hsa-miR-15b-5p	MIMAT0000417	41.06	73.45	40.97	30.27	190.25	99.72	73.65	89.10	46.44	113.18	1.28531	0.01948
hsa-miR-3127-5p	MIMAT0014990	16.76		11.45	11.84	38.56	22.72	19.15	30.61	13.35	27.76	1.05630	0.01965
hsa-let-7i-5p	MIMAT0000415	9.71	15.54	8.20	5.89	34.12	22.47	16.41	16.31	9.83	22.33	1.18283	0.02038
hsa-miR-575	MIMAT0003240	209.42	57.13	148.68	115.17	766.21	389.09	210.60	382.08	132.60	436.99	1.72056	0.02050
hsa-miR-6889-5p	MIMAT0027678	725.84	274.98	275.88	331.44	1253.14	899.72	643.90	740.65	402.03	884.35	1.13731	0.02096
hsa-miR-4505	MIMAT0019041	846.29	548.51	469.17	623.89	743.73	2132.86	1242.16	1512.25	621.96	1407.75	1.17849	0.02254
hsa-miR-1287-5p	MIMAT0005878	4.95		5.18	7.50	16.28	12.39	7.51	13.41	5.88	12.40	1.07669	0.02283
hsa-miR-6799-5p	MIMAT0027498	1192.15	382.64	505.90	632.82	2629.28	1504.55	1094.47	1335.01	678.38	1640.83	1.27426	0.02393
hsa-miR-4733-3p	MIMAT0019858	26.25	11.25	58.29	43.92	63.86	117.19	99.31	97.91	34.93	94.57	1.43691	0.02477
hsa-miR-331-5p	MIMAT0004700		11.92	11.38	8.98	3.22		5.46		10.76	4.34	-1.30972	0.02603
hsa-miR-433-5p	MIMAT0026554	30.79	12.23	14.09	22.00	146.11	45.81	35.23	42.84	19.78	67.50	1.77109	0.02754
hsa-miR-6075	MIMAT0023700	499.92	384.88	380.83	459.16	487.88	1374.86	743.36	890.61	431.20	874.18	1.01958	0.02851
hsa-miR-4740-3p	MIMAT0019870	118.26	41.95	62.39	60.49	357.49	144.87	111.85	139.62	70.77	188.46	1.41302	0.03080
hsa-miR-566	MIMAT0003230	8.55		13.27	6.47	13.10	25.69	17.99	18.66	9.43	18.86	1.00040	0.03117
hsa-miR-1202	MIMAT0005865	393.79	154.46	244.75	247.05	1344.20	529.77	383.64	491.35	260.01	687.24	1.40224	0.03617
hsa-miR-1343-5p	MIMAT0027038	5271.27	2472.80	2997.05	4726.10	17197.72	6993.22	5187.93	8411.36	3866.80	9447.56	1.28880	0.03656
hsa-miR-6734-5p	MIMAT0027369	34.54	14.55	15.47	18.49	72.62	42.35	28.97	32.41	20.76	44.09	1.08634	0.03886

hsa-miR-1273d	MIMAT0015090	4.84	8.10	7.59	7.53	26.41	12.23	7.79	17.17	7.02	15.90	1.18053	0.04062
hsa-miR-5585-3p	MIMAT0022286	201.59	61.29	73.91	205.49	609.75	215.90	221.01	437.78	135.57	371.11	1.45279	0.04301
hsa-miR-1275	MIMAT0005929	835.45	212.23	351.71	404.66	2302.14	954.17	655.41	910.82	451.01	1205.64	1.41855	0.04462
hsa-miR-186-3p	MIMAT0004612	14.31		6.71	4.93	13.47	22.14	19.19	14.86	8.65	17.41	1.00935	0.04608
hsa-miR-4746-5p	MIMAT0019880	20.84		13.00	29.34	11.08	4.59	6.65	12.94	21.06	8.82	-1.25632	0.04765
hsa-miR-125a-3p	MIMAT0004602	83.05	43.42	40.06	29.82	196.71	95.27	72.54	73.57	49.09	109.52	1.15775	0.04849
hsa-miR-223-3p	MIMAT0000280	112.86	44.32	162.24	46.86	198.22	354.04	219.22	118.65	91.57	222.53	1.28110	0.04901

Supplementary figure S1

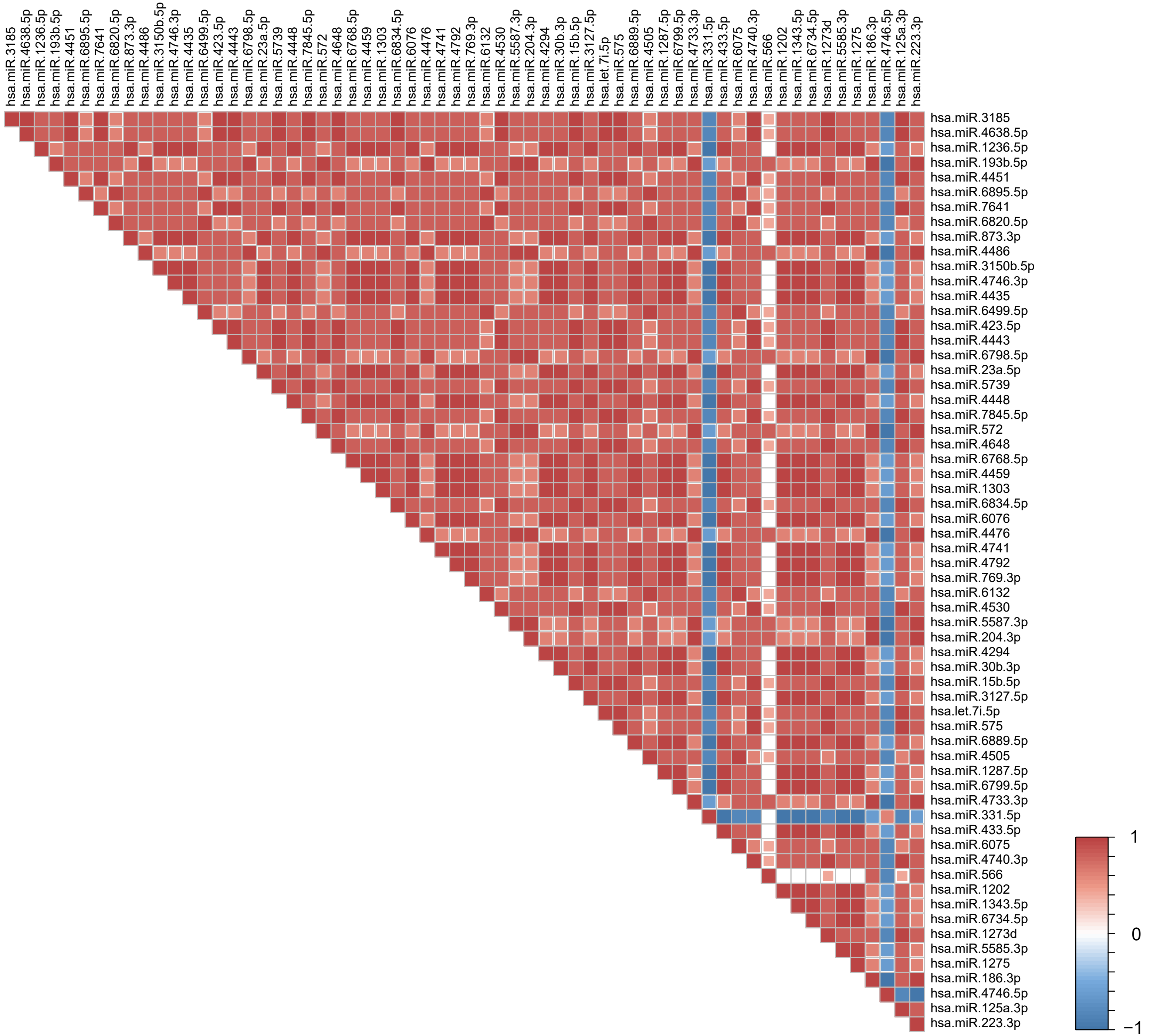

Supplement: Supplementary file 1 — Additional file 1: Table S1. Pathological criteria for the effects of chemotherapy. Table S2. The TaqMan® Assays used in this study. Table S3. Differentially expressed miRNA between responder and non-responder to neoadjuvant chemotherapy. Figure S1. Correlation analysis of expression levels of 62 miRNAs differentially expressed between responders to non-responders to neoadjuvant chemotherapy. [file 12967_2018_1762_MOESM1_ESM.pdf]
